# Growth Mindset and Older Adults’ Well-Being During the COVID-19 Pandemic

**DOI:** 10.1093/geroni/igab046.355

**Published:** 2021-12-17

**Authors:** Yena Kyeong, Pamela Sheffler, Esra Kürüm, Leah Ferguson, Elizabeth Davis, Carla Strickland-Hughes, Rachel Wu

**Affiliations:** 1 University of California, Riverside, Riverside, California, United States; 2 University of the Pacific, University of the Pacific, California, United States; 3 University Of California - Riverside, Riverside, California, United States

## Abstract

Growth mindset, the belief that abilities and attributes are improvable, may help buffer against older adults’ feelings of social isolation during the COVID-19 pandemic, as it may foster effective self-regulation and resilience. This study examined the effects of growth mindset on older adults’ well-being and adjustment, compared to younger and middle-aged adults. Participants self-reported on their growth mindset, depression, well-being, and daily habits amid the pandemic. For older adults (N = 178, 82% female, M age = 70.42, SD age = 6.50, range 60-90), regression analyses (controlling for gender, education, income, and age) revealed that growth mindset was associated with decreased depression (β = -.29, p = .001) and increased well-being (β = .38, p < .001). In addition, a logistic regression showed that older adults with a higher growth mindset were more likely to adjust their daily tasks during the pandemic (e.g., using technology to remotely socialize; OR = 1.77, p = .012). The same set of analyses in samples of younger (N = 235, 72% female, M age = 29.84, SD age = 5.89, range 18-39) and middle-aged adults (N = 188, 74% female, M age = 50.02, SD age = 6.10, range 40-59) revealed that growth mindset was associated with decreased depression and increased well-being. However, in these groups, growth mindset did not predict the likelihood of adjusting daily tasks. Findings suggest that while growth mindset is linked to enhanced well-being during the pandemic, its effect on adjusting to new circumstances might be salient in older adulthood.

